# Influence of tumor location on short- and long-term outcomes after laparoscopic surgery for rectal cancer: a propensity score matched cohort study

**DOI:** 10.1186/s12885-020-07255-9

**Published:** 2020-08-14

**Authors:** Hong Yang, Zhendan Yao, Ming Cui, Jiadi Xing, Chenghai Zhang, Nan Zhang, Maoxing Liu, Kai Xu, Fei Tan, Xiangqian Su

**Affiliations:** Key laboratory of Carcinogenesis and Translational Research (Ministry of Education), Department of Gastrointestinal Surgery IV, Peking University Cancer Hospital & Institute, 52 Fucheng Road, Haidian District, Beijing, 100142 PR China

**Keywords:** Low rectal cancer, Mid/high rectal cancer, Laparoscopic surgery, Oncological outcomes, Propensity score matching

## Abstract

**Background:**

This study aimed to evaluate the short- and long-term outcomes after laparoscopic resection for low rectal cancer (LRC) compared with mid/high rectal cancer (M/HRC).

**Methods:**

Patients with rectal cancer undergoing laparoscopic resection with curative intent were retrospectively reviewed between 2009 and 2015. After matched 1:1 by using propensity score analysis, perioperative and oncological outcomes were compared between LRC and M/HRC groups. Multivariate analysis was performed to identify independent factors of overall survival (OS) and disease-free survival (DFS).

**Results:**

Of 373 patients who met the criteria for inclusion, 198 patients were matched for the analysis. Laparoscopic surgery for LRC required longer operative time (P<0.001) and more blood loss volume (*P* = 0.015) compared with M/HRC, and the LRC group tended to have a higher incidence of postoperative complications (16.2% vs. 8.1%, *P* = 0.082). There was no significant difference in local recurrence between the two groups (9.1% vs. 4.0%, *P* = 0.251), whereas distant metastasis was inclined to be more frequent in LRC patients compared with M/HRC (21.2% vs. 12.1%, *P* = 0.086). The LRC group showed significantly inferior 5-year OS (77.0% vs. 86.4%, *P* = 0.033) and DFS (71.2% vs. 86.2%, *P* = 0.017) compared with the M/HRC group. Multivariate analysis indicated that tumor location was an independent predictor of DFS (HR = 2.305, 95% CI 1.203–4.417, *P* = 0.012).

**Conclusion:**

Tumor location of the rectal cancer significantly affected the clinical and oncological outcomes after laparoscopic surgery, and it was an independent predictor of DFS.

## Background

Rectal cancer is one of the most common malignant diseases worldwide. Nowadays, surgery remains the cornerstone for the treatment of rectal cancer. However, the treatment strategy for rectal cancer has changed dramatically in the past decades, for instance, the introduction of total mesorectal excision (TME), neoadjuvant chemoradiotherapy and minimally invasive surgery. Laparoscopic surgery for rectal cancer is widely performed all over the world in recent years. A number of clinical studies including some randomized clinical trials have confirmed that laparoscopic surgery was feasible and safe for rectal cancer, with favorable short-term benefits and similar oncological outcomes compared with open surgery [1–7]. Laparoscopic surgery for rectal cancer is considered to be a technically demanding procedure, especially for low rectal cancer. Some studies have already revealed that the tumor distance from the anal verge was related to the difficulty of laparoscopic surgery [8, 9]. Although several studies have explored the impact of rectal cancer height on clinical management and outcomes in patients undergoing curative resection, few studies have particularly compared the clinical and oncological outcomes of laparoscopic surgery at different heights of rectal cancer [10–13].

Therefore, the aim of the present study was to evaluate the influence of tumor location on short- and long-term outcomes of rectal cancer after laparoscopic resection with curative intent by propensity score analysis.

## Methods

### Patients

We retrospectively reviewed the records of all patients with histologically proven rectal adenocarcinoma who underwent laparoscopic resection with curative intent at the Department of Gastrointestinal Surgery IV, Peking University Cancer Hospital between 2009 and 2015. All the operations were performed laparoscopically by a same surgical team with rich experience in laparoscopic surgery. Patients with distant metastases, emergent surgery, palliative resection, combined evisceration, concurrent malignancies or a history of other malignancies within 5 years were excluded. This study was approved by the Research Ethics Committee of Peking University Cancer Hospital & Institute.

### Interventions

The treatment decision was based on the location and stage of the disease. All patients were evaluated by means of digital rectal examination, tumor marker levels (CEA, CA19–9 and CA72.4), chest radiography or computed tomography (CT), abdominal and pelvic CT, pelvic magnetic resonance imaging, and colonoscopy biopsy. In addition, endorectal ultrasonography was used in patients with low rectal cancer (LRC). LRC was defined as the lower edge of the tumor located less than 5 cm from the anal verge, while mid/high rectal cancer (M/HRC) as being above this level, which was assessed by preoperative colonoscopy.

Patients with locally advanced mid-low rectal cancer (defined as tumor located within 10 cm from the anal verge, with clinical stage ≥T3 or N+) were recommended to receive neoadjuvant therapy (NAT) in the form of long-course chemoradiotherapy (50.6 Gy in 22 fractions, 5 times per week over a month). Capecitabine (825 mg/m^2^ orally twice per day) was administered synchronously with radiotherapy. Surgery based on the principle of TME was performed within 6 to 10 weeks after the completion of radiotherapy, whereas patients without NAT underwent curative resection immediately.

The laparoscopic surgery was performed using five ports, same standardized principle and procedure were applied in most cases. The type of surgery either restorative or non-restorative was primarily depended on the distance of rectal cancer from the anal verge and the surgeon’s judgment during the operation. Restorative surgery was defined as low anterior resection (LAR). Non-restorative surgery included abdominoperineal resection (APR), extralevator abdominoperineal excision (ELAPE), and Hartmann’s procedure. Inferior mesenteric artery was divided proximal or distal to the left colic artery bifurcation, which was decided by the operating surgeon. Lymph node dissection was started near the origin of the inferior mesenteric artery. Mobilization of the sigmoid colon and rectum was required to comply with the principles of TME or partial mesorectal excision if the tumor was in the upper part of the rectum. Endoscopic linear staplers were used to divide the rectum to achieve a safe distal resection margin. The specimen was removed through a small incision using the port in the lower left quadrant or the anus. Transection of the bowel was performed extracorporeally. End-to-end anastomosis was then performed intracorporeally using the double stapling technique. Protective stoma was selectively performed according to tumor location and intraoperative conditions. APR or ELAPE was recommended when the levator muscle was invaded or preservation of the anus was impracticable. The perineal surgery and terminal colostomy were performed as described in the literature [14].

Pathologic evaluation was performed according to the American Joint Committee on Cancer TNM staging system (the seventh edition) [15]. Histopathologic results were independently reviewed by 2 pathologists. Positive circumferential resection margin (CRM) was defined as the distance from the specimen surface to the primary tumor or any tumor deposit ≤1 mm. About 4 weeks after surgery, patients with stage III or stage II disease with risk factors (poorly differentiated, peritoneal and serosal involvement, lymphovascular or perineural invasion, harvested lymph nodes less than 12 or positive CRM) were recommended to receive adjuvant chemotherapy (using mFOLFOX6, CapeOX or capecitabine alone) for 6 months. Adjuvant chemotherapy was also recommended for patients who received NAT.

### Follow-up

Patients were scheduled for follow-up every 3 months for the first 2 years after surgery, every 6 months for the next 3 years, and yearly thereafter. Follow-up examinations included a physical examination, complete blood cell count, blood biochemistry and serum CEA, CA19–9 and CA72.4 levels. Chest radiography or CT, abdominal and pelvic CT were performed every 6 months, and a colonoscopy was performed annually after the surgery. Local recurrence was defined as clinical, radiological, or pathologic evidence of malignancy near the site of surgical excision or draining lymph nodes. Distant metastasis was defined as recurrent disease in other organs. Overall survival (OS) was defined as the time from the day of surgery to that of death. Disease-free survival (DFS) was calculated from the day of surgery to that of any recurrence.

### Statistical analysis

Propensity score analysis was performed with SPSS (version 22.0, IBM Corporation, Chicago) including R-Essentials for SPSS and R version 2.15.3 software. Based on tumor location (LRC and M/HRC), patients were matched 1:1 by propensity score (nearest neighbor matching with logistic regression, caliper 0.2 without replacement) using the covariates age, sex, American Society of Anesthesiologists (ASA) score, body mass index (BMI), NAT, tumor differentiation, pathological T and N stage, TNM stage, lymphovascular invasion, perineural invasion, preoperative CEA, CA19–9 and CA72.4 levels.

Categorical variables were described as numbers with percentages and compared with either a chi-square or Fisher’s exact test. Continuous variables were expressed by median and range and analyzed using Mann-Whitney U test. OS and DFS were estimated using a Kaplan–Meier model, and comparisons were analyzed with the log-rank test. Parameters found to be associated with survival by the univariate analysis (based on a *P*-value <0.05) were entered into a multivariable Cox regression analysis. A *P*-value of <0.05 was considered statistically significant.

## Results

According to the inclusion and exclusion criteria, a total of 373 patients were enrolled in our analysis, including 138 patients in the LRC group and 235 patients in the M/HRC group. After propensity score matching at a ratio of 1:1 based on the variables mentioned above, 99 LRC patients were matched with 99 M/HRC patients.

### Characteristics and short-term outcome for the total cohort

The clinicopathologic characteristics of the patients are summarized in Table 1. In the total cohort, there were more elderly patients in the M/HRC group compared with the LRC group (*P* = 0.027). The pathological T stage and TNM stage of tumors were more advanced in the M/HRC group than in the LRC group (P<0.001 and *P* = 0.002). Perineural invasion happened more often in the M/HRC group (*P* = 0.009), while more patients received NAT in the LRC group (P<0.001). There were no statistical differences in the aspect of other clinicopathologic factors between the two groups.

**Table 1:** Demographic and pathological characteristics according to tumor location: overall cohort and matched cohort

Variables	Overall patients			Matched patients		
	LRC (*n* = 138)	M/HRC (*n* = 235)	*P* value	LRC (*n* = 99)	M/HRC (*n* = 99)	*P* value
Sex, n (%)			0.700			1.000
Male	78 (56.5)	128 (54.5)		52 (52.5)	52 (52.5)	
Female	60 (43.5)	107 (45.5)		47 (47.5)	47 (47.5)	
Age (y), n (%)			0.027			0.802
≤ 60	81 (58.7)	110 (46.8)		54 (54.5)	58 (58.6)	
>60	57 (41.3)	125 (53.2)		45 (45.5)	41 (41.4)	
ASA, n (%)			0.873			1.000
I	68 (49.3)	112 (47.7)		51 (51.5)	51 (51.5)	
II	57 (41.3)	97 (41.3)		39 (39.4)	39 (39.4)	
III	13 (9.4)	26 (11.1)		9 (9.1)	9 (9.1)	
BMI (kg/m^2^), n (%)			0.113			0.669
<25	73 (52.9)	144 (61.3)		52 (52.5)	55 (55.6)	
≥ 25	65 (47.1)	91 (38.7)		47 (47.5)	44 (44.4)	
Preoperative CEA (ng/ml), n (%)			0.374			0.644
≤ 5	100 (72.5)	160 (68.1)		67 (67.7)	70 (70.7)	
>5	38 (27.5)	75 (31.9)		32 (32.3)	29 (29.3)	
Preoperative CA19–9 (U/ml), n (%)			0.665			0.621
≤ 37	128 (92.8)	215 (91.5)		91 (91.9)	89 (89.9)	
>37	10 (7.2)	20 (8.5)		8 (8.1)	10 (10.1)	
Preoperative CA72.4 (U/ml), n (%)			0.311			0.267
≤ 6.7	121 (87.7)	197 (83.8)		85 (85.9)	90 (90.9)	
>6.7	17 (12.3)	38 (16.2)		14 (14.1)	9 (9.1)	
Tumor differentiation, n (%)			0.161			0.592
Well + moderate	111 (80.4)	202 (86.0)		81 (81.8)	78 (78.8)	
Poor	27 (19.6)	33 (14.0)		18 (18.2)	21 (21.2)	
Pathological T stage, n (%)			<0.001			0.461
pT0–2	64 (46.4)	66 (28.1)		39 (39.4)	34 (34.3)	
pT3–4	74 (53.6)	169 (71.9)		60 (60.6)	65 (65.7)	
Pathological N stage, n (%)			0.352			0.394
pN0	82 (59.4)	128 (54.5)		52 (52.5)	46 (46.5)	
pN1–2	56 (40.6)	107 (45.5)		47 (47.5)	53 (53.5)	
TNM stage, n (%)			0.002			0.606
0-I	52 (37.7)	51 (21.7)		31 (31.3)	25 (25.3)	
II	30 (21.7)	77 (32.8)		21 (21.2)	21 (21.2)	
III	56 (40.6)	107 (45.5)		47 (47.5)	53 (53.5)	
Lymphovascular invasion, n (%)	13 (9.4)	37 (15.7)	0.083	11 (11.1)	13 (13.1)	0.663
Perineural invasion, n (%)	2 (1.4)	18 (7.7)	0.009	2 (2.0)	1 (1.0)	1.000
Neoadjuvant therapy, n (%)	66 (47.8)	27 (11.5)	<0.001	30 (30.3)	25 (25.3)	0.428
Adjuvant chemotherapy, n (%)	80 (58.0)	117 (49.8)	0.126	59 (59.6)	64 (64.6)	0.464

*LRC* Low rectal cancer, *M/HRC* Mid/high rectal cancer, *ASA* American Society of Anesthesiologists, *BMI* Body mass index, *CEA* Carcinoembryonic antigen

Operative results of the patients are shown in Table 2. Of all cases, more patients underwent non-restorative surgery in the LRC group (P<0.001), and in patients receiving restorative surgery, more patients beared protective ostomy in the LRC group compared with the M/HRC group (P<0.001). Meanwhile, the LRC group had longer operative time and more blood loss volume than the M/HRC group (P<0.001). There were no significant differences between the two groups in terms of the distal resection margin, CRM status and conversion to open surgery, except for a smaller number of harvested lymph nodes in the LRC group (P<0.001). The overall morbidity in LRC patients was 17.4%, which was higher than M/HRC patients (10.2%, *P* = 0.046). However, there were no significant differences in reoperation rate and the length of postoperative hospital stay between the two groups. No mortality occurred in both groups.

**Table 2:** Perioperative outcomes and recurrence according to tumor location: overall cohort and matched cohort

Variables	Overall patients			Matched patients		
	LRC (*n* = 138)	M/HRC (*n* = 235)	*P value*	LRC (*n* = 99)	M/HRC (*n* = 99)	*P value*
Type of operation, n (%)			<0.001			<0.001
Restorative	41 (29.7)	228 (97.0)		32 (32.3)	95 (96.0)	
Non-restorative	97 (70.3)	7 (3.0)		67 (67.7)	4 (4.0)	
Operation time (range) (min)	231 (136–468)	182 (77–375)	<0.001	231 (136–435)	200 (77–375)	<0.001
Blood loss (range) (ml)	50 (10–300)	50 (5–2000)	<0.001	50 (10–300)	50 (5–600)	0.015
Protective ostomy in LAR, n (%)	36/41 (87.8)	106/228 (46.5)	<0.001	28/32 (87.5)	52/95 (54.7)	0.001
Harvested lymph nodes, n (%)			<0.001			0.437
<12	55 (39.9)	49 (20.9)		32 (32.3)	27 (27.3)	
≥12	83 (60.1)	186 (79.1)		67 (67.7)	72 (72.7)	
Distal resection margin (cm), n (%)			0.616			0.756
<1	6 (4.3)	13 (5.5)		5 (5.1)	6 (6.1)	
≥ 1	132 (95.7)	222 (94.5)		94 (94.9)	93 (93.9)	
Positive CRM, n (%)	4 (2.9)	3 (1.3)	0.431	3 (3.0)	3 (3.0)	1.000
Conversions, n (%)	2 (1.4)	8 (3.4)	0.334	1 (1.0)	4 (4.0)	0.369
Operative complications, n (%)	24 (17.4)	24 (10.2)	0.046	16 (16.2)	8 (8.1)	0.082
Reoperation, n (%)	6 (4.3)	3 (1.3)	0.082	4 (4.0)	1 (1.0)	0.369
Postoperative LOS (range) (days)	9 (5–26)	9 (3–33)	0.172	9 (5–26)	8 (3–33)	0.011
30-day mortality, n (%)	0 (0)	0 (0)	1.000	0 (0)	0 (0)	1.000
Recurrence, n (%)	35 (25.4)^a^	34 (14.5)^b^	0.009	28 (28.3)^a^	14 (14.1)^b^	0.015
Local recurrence	9 (6.5)	4 (1.7)	0.019	9 (9.1)	4 (4.0)	0.251
Distant metastasis	28 (20.3)	32 (13.6)	0.090	21 (21.2)	12 (12.1)	0.086
Lung	7 (5.1)	10 (4.3)	0.715	6 (6.1)	5 (5.1)	0.756
Liver	1 (0.7)	6 (2.6)	0.267	1 (1.0)	2 (2.0)	1.000
Other site	6 (4.3)	4 (1.7)	0.182	3 (3.0)	1 (1.0)	0.621
Multiple organs	14 (10.1)	12 (5.1)	0.065	11 (11.1)	4 (4.0)	0.104

*LRC* Low rectal cancer, *M/HRC* Mid/high rectal cancer, *LAR* Low anterior resection, *CRM* Circumferential resection margin, *LOS* Length of stay

^a/b^ Local recurrence and distant metastasis occurred synchronously in 2 cases both in LRC and M/HRC groups

### Short-term outcome for the matched cohort

After propensity score matching, there were no longer any significant differences between the LRC group and M/HRC group for most of the baseline characteristics, especially for age, pathological TNM stage, perineural invasion and whether receiving NAT (Table 1). Similar to the total cohort, more patients received non-restorative surgery in the LRC group compared with the M/HRC group (P<0.001). Of the 71 non-restorative procedures, 40 cases of APR, 24 cases of ELAPE and 3 cases of Hartmann’s procedure were performed in the LRC group, while 2 cases of APR, 1 case of ELAPE and 1 case of Hartmann’s procedure were performed in the M/HRC group. The LRC group demonstrated a statistically significant longer operative time (P<0.001) and more blood loss volume (*P* = 0.015) when compared with the M/HRC group. There were no significant differences between the two groups in terms of the distal resection margin, CRM status, number of harvested lymph nodes and conversion to open surgery, except for more frequent protective ostomy in the LRC group (*P* = 0.001). The LRC patients were inclined to have more postoperative morbidity compared with M/HRC patients (16.2% vs. 8.1%, *P* = 0.082), which may prolong the length of hospital stay for LRC patients to some extent (*P* = 0.011). However, the reoperation rate and 30-day mortality were statistically insignificant between the two groups (Table 2).

### Long-term outcome for the matched cohort

**Fig. 1:**
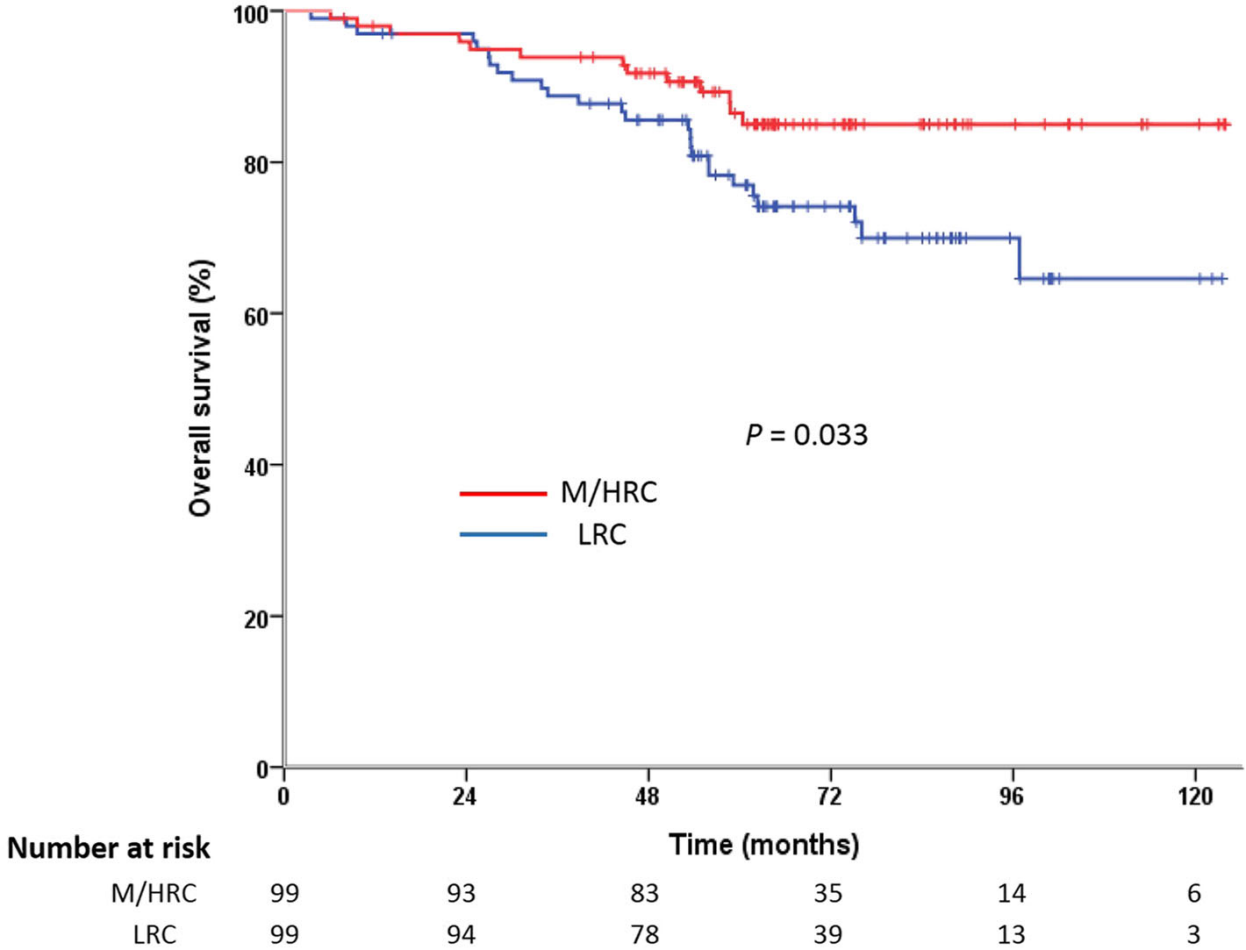
Kaplan-Meier curves of overall survival for patents of LRC vs. M/HRC

**Fig. 2:**
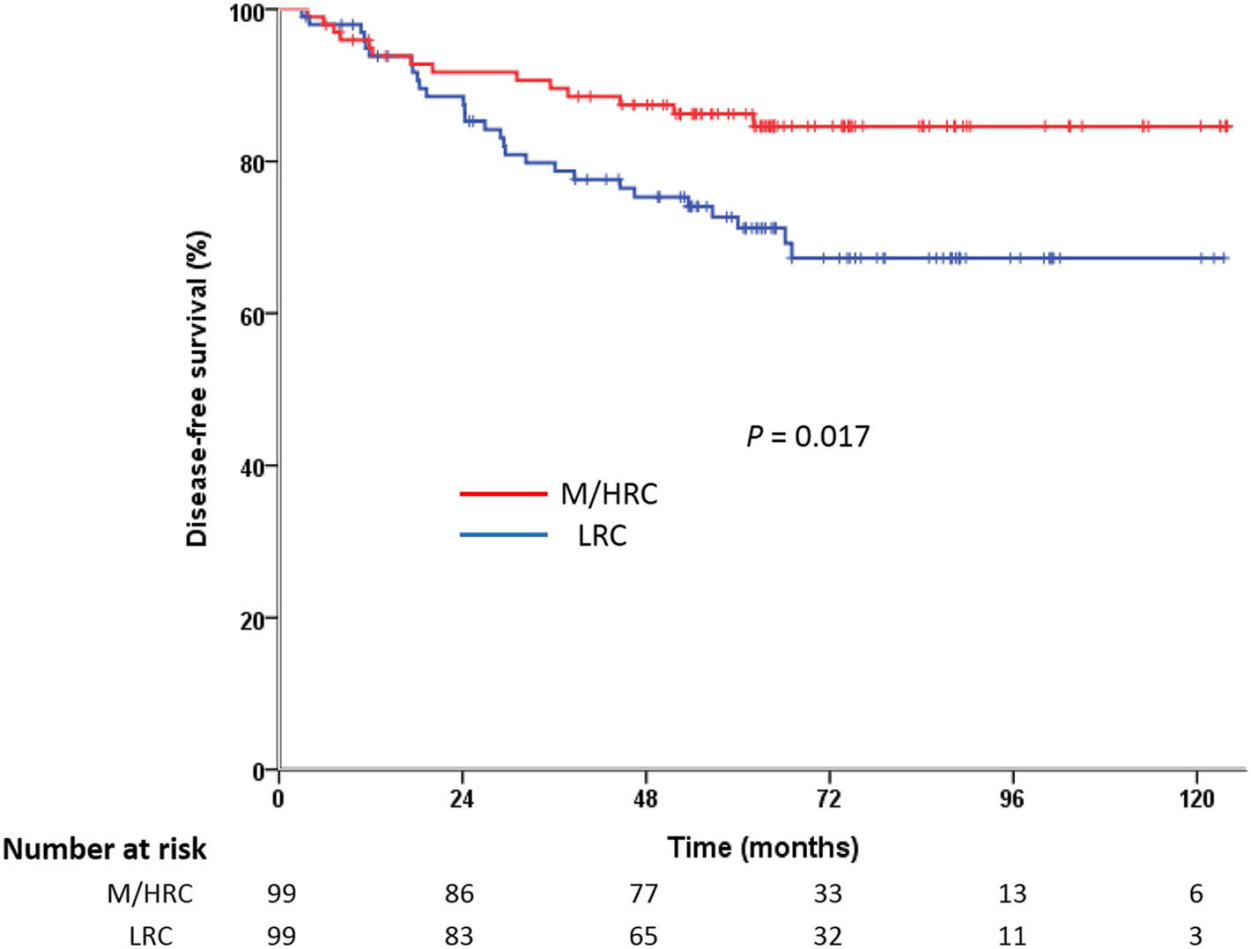
Kaplan-Meier curves of disease-free survival for patents of LRC vs. M/HRC

The median follow-up period was 63 months (range, 4–124 months) for the matched cohort. Recurrence was observed in 42 patients: 9 had local recurrence, 29 had distant metastasis and 4 developed local and distant recurrence synchronously. As a whole, recurrence was more frequent in the LRC group compared with the M/HRC group. Separately, the LRC patients tended to have a higher risk of distant metastasis than M/HRC patients (21.2% vs. 12.1%, *P* = 0.086). However, the incidence of local recurrence was 9.1% in the LRC group and 4.0% in the M/HRC group, which was statistically insignificant (*P* = 0.251). The patterns of recurrence are described in detail in Table 2. On Kaplan-Meier analysis, the 5-year OS was 77.0% for LRC patients and 86.4% for M/HRC patients (*P* = 0.033, Fig. 1); the 5-year DFS was 71.2 and 86.2%, respectively (*P* = 0.017, Fig. 2).

Based on univariate analysis, age (*p* = 0.001), tumor location (*P* = 0.033), preoperative CEA level (*P* = 0.043), preoperative CA19–9 level (*P* = 0.006), pathological T stage (*p* = 0.008), N stage (P<0.001), lymphovascular invasion (P<0.001) and postoperative complications (*P* = 0.003) were revealed as significant predictors of OS (Table 3). On multivariate analysis, only age (HR = 4.236, 95% CI 1.915–9.368, P<0.001), pathological N stage (HR = 5.006, 95% CI 1.874–13.368, *P* = 0.001) and lymphovascular invasion (HR = 3.086, 95% CI 1.368–6.960, *P* = 0.007) remained as independent factors of OS (Table 4).

**Table 3:** Univariate analysis of prognostic factors for overall survival (OS) and disease-free survival (DFS) in matched cohort

Variables	Numbers	5-year OS (%)	*P* value	5-year DFS (%)	*P* value
Sex					
Male	104	83.4	0.607	84.4	0.057
Female	94	79.4		72.4	
Age(y)					
≤60	112	89.7	0.001	84.8	0.010
>60	86	71.3		70.3	
ASA					
I	102	84.9	0.293	84.4	0.036
II- III	96	78.3		72.3	
BMI (kg/m^2^)					
<25	107	77.3	0.074	79.7	0.986
≥25	91	86.7		77.7	
Location					
Mid/high	99	86.4	0.033	86.2	0.017
Low	99	77.0		71.2	
Preoperative CEA (ng/ml)					
≤5	137	82.6	0.043	82.2	0.141
>5	61	79.1		71.4	
Preoperative CA19–9 (U/ml)					
≤37	180	84.0	0.006	80.6	0.019
>37	18	58.8		60.2	
Preoperative CA72.4 (U/ml)					
≤6.7	175	82.2	0.543	79.1	0.436
>6.7	23	78.0		75.6	
Tumor differentiation					
Well/moderate	159	84.3	0.079	79.1	0.915
Poor	39	71.0		76.9	
Pathological T stage					
pT0–2	73	92.0	0.008	85.2	0.288
pT3–4	125	75.6		74.7	
Pathological N stage					
pN0	98	94.2	<0.001	86.0	0.026
pN1–2	100	68.9		71.2	
Lymphovascular invasion					
Negative	174	86.7	<0.001	80.3	0.118
Positive	24	46.7		65.5	
Perineural invasion					
Negative	195	82.0	0.192	79.0	0.200
Positive	3	50.0		50.0	
Type of operation					
Restorative	127	86.2	0.225	81.3	0.235
Non-restorative	71	73.5		73.8	
Harvested lymph nodes					
≥12	139	82.4	0.632	82.6	0.018
<12	59	80.2		69.6	
Distal resection margin (cm)					
≥1	187	81.7	0.871	77.5	0.096
<1	11	80.8		100.0	
CRM (mm)					
>1	192	81.7	0.310	80.2	<0.001
≤1	6	80.0		33.3	
Operative complications					
No	174	84.8	0.003	79.7	0.569
Yes	24	59.5		71.9	
Reoperation					
No	193	82.3	0.121	78.1	0.263
Yes	5	60.0		100.0	
Neoadjuvant therapy					
No	143	82.4	0.519	81.9	0.035
Yes	55	80.2		70.2	
Adjuvant chemotherapy					
No	82	79.2	0.882	82.4	0.310
Yes	116	83.3		76.2	

*ASA* American Society of Anesthesiologists, *BMI* Body mass index, *CEA* Carcinoembryonic antigen, *CRM* Circumferential resection margin

**Table 4:** Multivariate analysis of prognostic factors for overall survival (OS) and disease-free survival (DFS) in matched cohort

Variables	OS			DFS		
	HR	95% CI	*P* value	HR	95% CI	*P* value
Age(y)			<0.001			0.172
≤ 60	1			1		
>60	4.236	1.915–9.368		1.606	0.813–3.170	
ASA			–			0.071
I				1		
II- III				1.952	0.943–4.040	
Location			0.099			0.012
Mid/high	1			1		
Low	1.801	0.894–3.629		2.305	1.203–4.417	
Preoperative CEA (ng/ml)			0.908			–
≤ 5	1					
>5	0.954	0.431–2.110				
Preoperative CA19–9 (U/ml)			0.901			0.046
≤ 37	1			1		
>37	1.061	0.420–2.680		2.362	1.014–5.505	
Pathological T stage			0.340			–
pT0–2	1					
pT3–4	1.579	0.618–4.035				
Pathological N stage			0.001			0.010
pN0	1			1		
pN1–2	5.006	1.874–13.368		2.438	1.239–4.797	
Lymphovascular invasion			0.007			–
Negative	1					
Positive	3.086	1.368–6.960				
Harvested lymph nodes			–			0.093
≥ 12				1		
<12				1.955	0.895–4.270	
CRM (mm)			–			<0.001
>1				1		
≤ 1				8.609	2.826–26.228	
Operative complications			0.573			–
No	1					
Yes	1.267	0.556–2.890				
Neoadjuvant therapy			–			0.476
No				1		
Yes				1.333	0.605–2.937	

- Variable not included in multivariate analysis

*ASA* American Society of Anesthesiologists, *CEA* Carcinoembryonic antigen, *CRM* Circumferential resection margin, *HR* Hazard ratio, *CI* Confidence interval

Considering the DFS, univariate analysis revealed age (*P* = 0.010), ASA score (*P* = 0.036), tumor location (*P* = 0.017), preoperative CA19–9 level (*P* = 0.019), pathological N stage (*P* = 0.026), number of harvested lymph nodes (*P* = 0.018), CRM status (*P*<0.001) and neoadjuvant CRT (*P* = 0.035) as significant predictors of DFS (Table 3). On multivariate analysis, only tumor location (HR = 2.305, 95% CI 1.203–4.417, *P* = 0.012), preoperative CA19–9 level (HR = 2.362, 95% CI 1.014–5.505, *P* = 0.046), pathological N stage (HR = 2.438, 95% CI 1.239–4.797, *P* = 0.010) and CRM status (HR = 8.609, 95% CI 2.826–26.228, P<0.001) were independent predictors of DFS (Table 4).

## Discussion

Over the past few decades, minimally invasive surgery has been introduced into the treatment of rectal cancer, and more excisions were performed laparoscopically. Our center is one of the earliest medical institutions to carry out laparoscopic resection of rectal cancer in China. In the present study, laparoscopic surgery for LRC required longer operative time and more blood loss volume than M/HRC. The main reason for this was that the difficulty of laparoscopic resection for LRC may be increased due to narrow space and complex anatomy at the bottom of the pelvic, and requiring more non-restorative surgery or protective ostomy during operation. Though a trend to higher overall morbidity was observed in the LRC group compared with the M/HRC group, which may prolong the duration of postoperative hospital stay, the reoperation rate and 30-day mortality did not increase. Akiyoshi et al. [8] demonstrated that the tumor distance from the anal verge was one of the independent predictors of pelvic operative time and postoperative morbidity. Ogiso et al. [9] also concluded that tumor location was an independent predictor of operative time, which was related to intraoperative blood loss.

In addition, our results showed that 5-year OS and DFS rates were poorer for LRC patients compared with M/HRC patients after laparoscopic surgery, which is consistent with previous literatures [10, 14], although these studies were not focused exclusively on laparoscopic surgery. Chiang et al. [10] noted that the rectal cancer level significantly affected the long-term survival and patterns of distant metastases for patients who underwent surgical resection. Compared with mid-rectal and upper-rectal cancers, LRC had the worst prognosis. Cheng et al. [14] divided T3/T4 rectal cancer patients who underwent surgery into high and mid/low rectal cancer, they found that patients with stage III high rectal cancer demonstrated better prognosis than those with mid/low rectal cancer, and tumor location was an independent prognostic factor for long-term survival. However, other studies have come to different conclusions, meaning that tumor location has no influence on long-term outcome. Bhangu et al. [11] concluded that low height of rectal cancer after curative surgery did not lead to worse survival, LRC showed equivalent oncological outcome compared with M/HRC. Similarly, Khan et al. [12] also found that although the level of rectal cancer affected the use of NAT and R0 resection rate, it did not affect recurrence rate and long-term survival.

In our matched cohort, the pathological TNM stage of LRC and M/HRC patients was nearly equal, so the long-term survival of the two groups was more comparable. Considering there were more patients of LRC received non-restorative surgery compared with M/HRC, and this may have some impact on long-term survival, multivariate analysis was carried out. On multivariate analysis, we came to the conclusion that tumor location remained as an independent predictor for PFS, but not for OS. When comparing the total recurrence between the two groups, LRC patients had higher risk of tumor relapse. This may explain why LRC patients had worse long-term survival than M/HRC patients. However, when analyzed separately, there was no significant difference in the local recurrence rate between the two groups, though the distant metastasis rate tended to be higher in LRC patients. Considering the small number of patients with recurrence in the series, this finding should be regarded with caution. Cheng et al. [14] found that the location of rectal cancer was a significant risk factor for local recurrence, lung metastasis, bone metastasis and systemic lymph node metastasis, as the tumor distance from the anal verge decreased, the risk for recurrence significantly increased. Frambach et al. [16] retrospectively analyzed 378 patients with locally advanced rectal cancer treated with NAT and curative surgery. They concluded that a distance of the tumor from the anal verge ≤5 cm was the risk factor for recurrence, and it was the only factor associated with increased risk of lung metastasis. Since LRC is more prone to distant metastasis after surgery, its perioperative treatment should be strengthened. At present, preoperative radiotherapy combined with intensive chemotherapy for advanced rectal cancer is one of the research hotspots. In addition, some studies have shown that adjuvant chemotherapy is still necessary for patients with obvious tumor downstaging after NAT, even if complete pathological response has been achieved [17, 18].

The results of this study showed that overall positive CRM rate in the matched cohort was lower (3.0%) than most previous studies have reported [3, 19, 20], and did not differ significantly between LRC and M/HRC patients. The COLOR II study [3, 4] presented that positive CRM rate was 10% after laparoscopic surgery for rectal cancer, and the rate of local recurrence was 5% at 3 years. The presence of involved CRM after laparoscopic surgery in the ALaCaRT [19] and the ACOSOG Z6051 [20] randomized clinical trials was 12 and 7%, respectively. While some other studies have reported relatively low positive CRM rates. The COREAN study [1] noted that CRM positivity was 2.9% after laparoscopic surgery for mid/low rectal cancer after NAT. Park et al. [21] demonstrated that positive CRM rate was 2.3% after laparoscopic intersphincteric resection for low rectal cancer, and 3-year local recurrence rate was 2.6%. We speculated the main reason for the low rate of positive CRM in our research was that pathologists may have underestimated the rate of CRM involvement. Besides this, all the operations in this study were performed by a same surgical team and may be related to this result, because in this case surgical standards and procedures were easily unified to ensure high quality of operations. However, considering the postoperative local recurrence rate was 9.1 and 4.0% for LRC and M/HRC patients, respectively, we deem the actual positive CRM rate would be a little higher than observed in this group.

Compared with M/HRC, LRC was more likely to harvest less than 12 lymph nodes in the total cohort. The proportion of dissected lymph nodes less than 12 in the two groups was 39.9 and 20.9%, respectively. This result can be explained by the fact that more patients in the LRC group had received NAT, which accounted for 47.8 and 11.5% for LRC and M/HRC patients, respectively. Several studies have shown that NAT was frequently associated with decreased number of harvested lymph nodes, regardless of the application of TME principle and appropriate pathologic evaluation [22–25]. Moreover, some of the findings also noted that retrieval of less than 12 lymph nodes in the proctectomy specimen of rectal cancer treated with NAT had no adverse effect on long-term survival and may be a marker of higher tumor response [26–29]. On the contrary, other studies indicated that lymph node yield was an independent predictor of survival in rectal cancer irrespective of NAT [30, 31]. In our matched cohort, propensity score matching basically eliminated the difference in NAT between LRC and M/HRC patients. The number of harvested lymph nodes were equivalent and the results were comparable between the two groups. The univariate analysis showed that harvested lymph nodes less than 12 was an inferior predictor of PFS, which should be taken seriously.

To our knowledge, this is the only study comparing the short- and long-term outcomes of laparoscopic surgery for LRC and M/HRC by propensity score analysis. However, the present study has a few limitations, such as retrospective design and small samples which introduce inherent selection bias and limit the generalizability of the results. Furthermore, in this study, we divided rectal cancer into LRC and M/HRC, mainly referring to the grouping approach of previous literatures [11, 12]. From a surgical point of view, laparoscopic surgery for low rectal cancer is considered to be more challenging, with higher rates of positive CRM and permanent stoma, whereas the surgical procedures and difficulties of mid and high rectal cancer are more alike. However, this grouping method has some limitations, especially the perioperative treatment is different depending on tumor location, and NAT is more often recommended for mid/low rectal cancer but less for high rectal cancer. Finally, due to the limitations of patient compliance and economic condition, the treatment options for the cases included in this study were not always reasonable. For example, some patients who required NAT accepted surgery immediately, and other patients who were recommended for adjuvant chemotherapy refused it.

## Conclusion

In conclusion, our study showed that tumor location of the rectal cancer significantly affected the clinical and oncological outcomes after laparoscopic surgery. Lower level of the rectal cancer was related to longer operative time and more blood loss volume, and inclined to have higher postoperative morbidity. Patients of LRC presented significantly inferior OS and DFS, and tended to develop more distant metastasis compared to M/HRC. Besides, tumor location was an independent predictor of DFS for rectal cancer after laparoscopic surgery.

## Data Availability

The data that support the findings of this study are available on request from the corresponding author. The data are not publicly available due to privacy or ethical restrictions.

## Abbreviations

LRC: Low rectal cancer
M/HRC: Mid/high rectal cancer
OS: Overall survival
DFS: Disease-free survival
TME: Total mesorectal excision
CEA: Carcinoembryonic antigen
CT: Computed tomography
NAT: Neoadjuvant therapy
LAR: Low anterior resection
APR: Abdominoperineal resection
ELAPE: Extralevator abdominoperineal excision
CRM: Circumferential resection margin
ASA: American Society of Anesthesiologists
BMI: Body mass index
